# An Enlarged and Infected Prostatic Utricle as a Rare Cause of Lower Urinary Tract Symptoms in Adolescent Males

**DOI:** 10.1155/2021/5516232

**Published:** 2021-06-18

**Authors:** Rashida Shakir, Michael G. Packer, Zarine R. Balsara

**Affiliations:** ^1^Children's Hospital of Philadelphia Care Network at Virtua, Voorhees, New Jersey, USA; ^2^Perelman School of Medicine, University of Pennsylvania, Philadelphia, Pennsylvania, USA; ^3^Urology for Children, Virtua Hospital, Voorhees, New Jersey, USA

## Abstract

Dysuria with lower abdominal pain is a common presentation for a urinary tract infection (UTI), and diagnosis is based on symptoms together with a urinalysis and urine culture suggestive of infection. UTI is uncommon in circumcised males who are not sexually active. When urine culture is negative, alternate diagnoses including, but not limited to, gastroenteritis, severe constipation, appendicitis, or epididymitis need to be considered. In patients with a known urologic history of proximal hypospadias and/or disorders of sexual development, rarer diagnoses also need to be considered. This paper reports the case of a 13-year-old male with a remote history of proximal hypospadias repair, who presented with nonspecific lower urinary tract symptoms. Initially he was treated for UTI. However, urine cultures remained negative despite persistent urinary tract symptoms. On further workup, he was found to have an enlarged and infected prostatic utricle. This report illustrates the importance of considering an enlarged prostatic utricle in the differential diagnoses of patients with chronic lower urinary tract symptoms and a history of hypospadias. Additionally, this case highlights the utility of magnetic resonance imaging (MRI) in clarifying lower urinary tract anatomy in cases where ultrasound is inconclusive.

## 1. Introduction

A prostatic utricle is a rudimentary structure in males that is a remnant of the fused caudal ends of the Mullerian ducts. An enlarged prostatic utricle is historically associated with proximal hypospadias and/or disorders of sexual development, although it has been reported in males without any external genital anomalies [1]. The majority of prostatic utricles are asymptomatic when small and often go undiagnosed. Symptoms occur if urine and debris are trapped in the pouch causing progressive enlargement with mass effects. Symptoms typically occur in the first or second decade of life. As the symptoms are suggestive of the more common scenario of urinary tract infection, misdiagnosis is common. Therefore, a high index of suspicion is required in males with a history of proximal hypospadias repair who present with lower urinary tract symptoms and a negative urine culture.

We report the case of a 13-year-old male with a history of mixed gonadal dysgenesis and proximal hypospadias repair as an infant who presented with dysuria, abdominal pain, and cloudy urine. Diagnosis was delayed due to the similarity in symptoms with UTI and due to the dilated prostatic utricle being mistaken as a dilated rectum on ultrasound. MRI was ultimately helpful in establishing the correct diagnosis. Physicians should be aware of the association between proximal hypospadias and an enlarged prostatic utricle and its presentation as recurrent UTI. Treatment is indicated for symptomatic prostatic utricles.

## 2. Case Report

A 13-year-old male presented to the pediatric emergency department (ED) of a community hospital with lower abdominal pain and pain with urination for 2 days. He had occasional vomiting and loose stools. He had a fever 1 day prior to presentation. In the ED, he had a low-grade fever, a mildly distended abdomen, and lower abdominal tenderness. Urine was turbid with too numerous to count white blood cells (WBCs). He denied sexual activity. His past medical and surgical history was notable for a two-stage proximal hypospadias repair as an infant, which was complicated by the development of a urethrocutaneous fistula. The fistula was subsequently repaired at 3 years of age. He also had a history of mixed gonadal dysgenesis and had a left orchiectomy for a left streak gonad with fallopian tube remnants as an infant. A urine culture was sent, and he was discharged on oral cephalexin with a presumptive diagnosis of UTI.

He returned to the ED 2 days later for persistent pain at the end of urination and lower abdominal pain. There were no interval fever or chills, and diarrhea had resolved. He reported worsening suprapubic and right lower quadrant pain, dysuria, urinary urgency and frequency, and foul-smelling urine. He had decreased appetite and activity. Stools had been small and clumped recently, but he denied chronic constipation. His mother reported a small amount of urethral discharge seen on the toilet paper used for wiping after urination. In the ED, he was afebrile and had lower abdominal tenderness on palpation. Genitourinary exam was notable for a circumcised penis, absent left testis, and descended right testis that was mildly tender to palpation but had a normal lie and cremasteric reflex. Urine culture from the prior ED visit showed no growth, although repeat urinalysis at the current visit still showed too numerous to count WBCs. The peripheral WBC count was normal, and C-reactive protein was elevated at 20 mg/dL.

**Figure 1:**
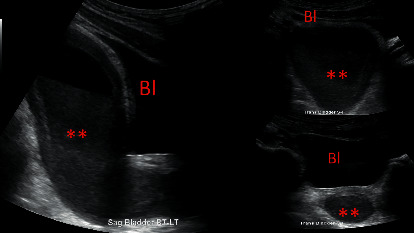
Our patient's ultrasound shows an enlarged fluid-filled structure (^*∗∗*^) filled with debris which is located in the midline and posterior to the bladder (Bl).

Scrotal ultrasound showed a normal right testicle with normal flow and no concerning pathology. Abdominal ultrasound could not visualize the appendix in its entirety, but no secondary signs of appendicitis were seen. Retroperitoneal ultrasound showed normal bladder and kidneys. The radiologist reported circumferential wall thickening of the rectum with an air-fluid level (Figure 1).

**Figure 2:**
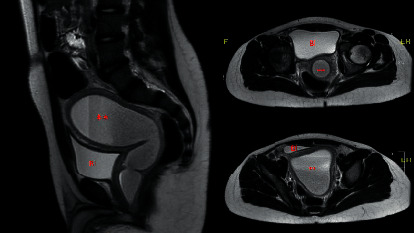
Our patient's MRI of the abdomen and pelvis (sagittal and axial) more clearly demonstrates that the fluid-filled structure located in the midline and posterior to the bladder (Bl) is actually an enlarged prostatic utricle (^*∗∗*^) filled with debris.

The differential diagnosis included UTI, appendicitis, scrotal pathology, severe constipation, or gastroenteritis. Scrotal pathology was ruled out by ultrasound. A urine culture had shown no growth, arguing against a bacterial urinary tract infection. Viral UTI was considered unlikely in the absence of significant pyuria. Symptoms of diarrhea, vomiting, and abdominal pain suggested a possible gastroenteritis. The circumferential wall thickening of the rectum with an air-fluid level would make gastroenteritis a reasonable diagnosis. However, the presence of significant pyuria and dysuria would be unusual. The abdominal ultrasound did not fully visualize the appendix. While the absence of secondary signs of appendicitis was reassuring, appendicitis could not be ruled out. To help elucidate any abdominal pathology contributing to his symptoms, MRI of the abdomen and pelvis with contrast was performed. It showed a large, smooth, thick-walled, fluid-filled structure within the pelvis that measured 13.9 × 9.1 × 7.3 cm and appeared to arise from the urethra. The structure was consistent with an enlarged prostatic utricle, which is a Mullerian duct remnant. Comparison with the retroperitoneal ultrasound suggested that the presumed fluid-filled rectum was actually the enlarged prostatic utricle located posterior to the bladder (Figures 1 and 2).

Further review of the patient's past medical history was revealing. Following penoscrotal hypospadias repair as an infant, he had recurrent UTI. Voiding cystourethrogram (VCUG) was performed and showed no vesicoureteral reflux. However, on the postvoid film, there was a round structure projecting over the lower right pelvis of uncertain etiology. In hindsight, this was likely the prostatic utricle. He developed an urethrocutaneous fistula following his hypospadias repair. At the time of urethrocutaneous fistula repair about 1 year later, a cystourethroscopy was performed, but the neck of the prostatic utricle was not visualized at the time.

He was admitted to the pediatric unit and started on IV ceftriaxone. Pediatric urology was consulted, and he was taken to the operating room the following day. Cystourethroscopy was performed and demonstrated debris within the bladder. A small utricular opening was visualized with efflux of debris. The utricular orifice was cannulated, and aspiration yielded 160 mL of thick, mucinous, foul-smelling fluid that was sent for culture. A generous incision of the orifice was performed to allow for adequate drainage of the utricular contents per urethra. He tolerated the procedure well. Culture of the aspirated fluid grew multiple organisms, whereas intraoperative urine culture showed no growth. He was discharged on oral Augmentin. He did well, but symptoms recurred. Therefore, he underwent elective robotic-assisted laparoscopic excision of the prostatic utricle several months later at a tertiary-care center.

## 3. Discussion

A prostatic utricle is a rudimentary structure that arises at the level of the verumontanum in the posterior urethra between the openings of the ejaculatory ducts and is present to some extent in all males. Traditional thinking is that the prostatic utricle is a remnant of the fused caudal ends of the Mullerian ducts that regress in males. This view has more recently been challenged by histologic evidence suggesting that the prostatic utricle may derive, at least partially, from the urogenital sinus [1–3]. An enlarged utricle is historically associated with proximal hypospadias, cryptorchidism, and/or intersex conditions but may be present with normal external genitalia [1, 2]. There is an increased likelihood of finding an enlarged utricle with increasing severity of proximal hypospadias.

A majority of prostatic utricles may go undiagnosed, as they are asymptomatic when small. When symptoms do occur, they typically present within the first or second decade of life. Symptoms occur if urine and debris are trapped in the pouch causing progressive enlargement with mass effects on the urethra, urinary bladder, or ureterovesical junction. The contents may get infected with bacteria. The most common symptoms of a prostatic utricle are dysuria, urinary retention, epididymitis, hematuria, and urinary incontinence. As these symptoms are suggestive of the more common scenario of urinary tract infection, misdiagnosis is common [1, 2].

There are several case reports of enlarged prostatic utricles in the literature [4–6]. Enlarged prostatic utricles need to be differentiated from Mullerian duct cysts, which do not communicate with the urethra and are not typically associated with other genitourinary anomalies. Diagnostic suspicion for an enlarged prostatic utricle may be raised by a cystic mass on digital rectal exam although this exam is not commonly performed in the pediatric population. Ultrasound (pelvic, transrectal, or perineal), VCUG, or retrograde urethrogram are used to confirm the diagnosis. Computerized tomography or MRI is helpful when the diagnosis is uncertain. Utricles are classified as grade 0, I, II, or III based on location of the opening and size of the cavity. Urinary stasis within the prostatic utricle can lead to the uncommon complication of intraluminal calculus formation [7]. Neoplasia can occur within the utricle in rare cases [8, 9].

Treatment of prostatic utricles is recommended only for symptomatic lesions. Treatment of acute symptoms may comprise antimicrobial therapy as well as drainage of cyst contents. Options for drainage include transperineal or transrectal aspiration or cystourethroscopy with catherization and dilation of the utricular orifice. However, these approaches rarely afford definitive treatment. When definitive surgical treatment is indicated, open, laparoscopic, or robotic-assisted laparoscopic excision of the prostatic utricle is performed [1, 2]. Due to the close proximity of the prostatic utricle to the vas deferens, pelvic nerves, and ejaculatory ducts, invasive surgery carries the risk of impotency and infertility if these structures are damaged during dissection. Definitive treatment of prostatic utricles carries a risk and should be reserved for those who are symptomatic and/or have failed percutaneous or endoscopic drainage.
